# Crystal structure of (*S*)-1-(1,3-benzo­thia­zol-2-yl)-2,2,2-tri­fluoro­ethanol

**DOI:** 10.1107/S1600536814016547

**Published:** 2014-08-01

**Authors:** Svitlana V. Shishkina, Olexandr V. Kucher, Anastasiya O. Kolodiazhnaya, Oleg B. Smolii, Andrey A. Tolmachev

**Affiliations:** a STC "Institute for Single Crystals", National Academy of Sciences of Ukraine, 60 Lenina ave., Kharkiv 61001, Ukraine; b Institute of Bioorganic Chemistry and Petrochemistry, National Academy of Science of Ukraine, 1 Murmanska St, Kyiv 02904, Ukraine; c ChemBioCenter, Kyiv National Taras Shevchenko University, 61 Chervonotkatska St, Kyiv 02094

**Keywords:** crystal structure, 1,3-benzo­thia­zole, 2,2,2-tri­fluoro­ethanol, hydrogen bonding

## Abstract

In the title compound, C_9_H_6_F_3_NOS, the 1,3-benzo­thia­zole ring system is essentially planar, with an r.m.s. deviation of 0.006 Å. In the crystal, mol­ecules are linked *via* O—H⋯N hydrogen bonds, forming zigzag chains along [010].

## Related literature   

For the synthesis of 1-substituted 2,2,2-tri­fluoro­ethanols from ketones, see: Yamazaki *et al.* (1993 ▶). For the enzymatic kinetic resolution of 1-substituted 2,2,2-tri­fluoro­ethanols, see: Omote *et al.* (2001 ▶); Xu *et al.* (2009 ▶). For the utilization of cinchonidine as a chiral solvating reagent, see: Kolodyazhnyi *et al.* (2006 ▶).
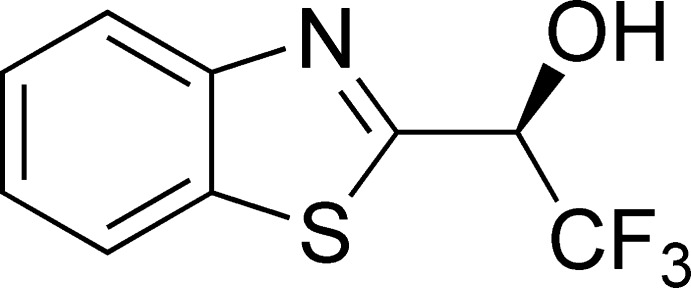


## Experimental   

### Crystal data  

C_9_H_6_F_3_NOS
*M* *_r_* = 233.21
Monoclinic, 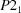

*a* = 9.2116 (9) Å
*b* = 5.5052 (4) Å
*c* = 10.2279 (8) Å
β = 107.411 (9)°
*V* = 494.91 (7) Å^3^
*Z* = 2
Mo *K*α radiation
μ = 0.34 mm^−1^
*T* = 293 K
0.20 × 0.05 × 0.05 mm

### Data collection  

Agilent Xcalibur3 diffractometer
Absorption correction: multi-scan (*CrysAlis RED*; Agilent, 2012 ▶) *T*_min_ = 0.935, *T*_max_ = 0.983
4650 measured reflections
2768 independent reflections
2293 reflections with *I* > 2σ(*I*)
*R*_int_ = 0.027

### Refinement  

*R*[*F* ^2^ > 2σ(*F* ^2^)] = 0.042
*wR*(*F* ^2^) = 0.108
*S* = 1.12
2768 reflections
160 parameters
4 restraints
All H-atom parameters refined
Δρ_max_ = 0.21 e Å^−3^
Δρ_min_ = −0.20 e Å^−3^
Absolute structure: Flack (1983 ▶), 1199 Friedel pairs
Absolute structure parameter: −0.03 (9)

### 

Data collection: *CrysAlis CCD* (Agilent, 2012 ▶); cell refinement: *CrysAlis CCD*; data reduction: *CrysAlis RED* (Agilent, 2012 ▶); program(s) used to solve structure: *SHELXTL* (Sheldrick, 2008 ▶); program(s) used to refine structure: *SHELXTL*; molecular graphics: *SHELXTL*; software used to prepare material for publication: *SHELXTL*.

## Supplementary Material

Crystal structure: contains datablock(s) I, global. DOI: 10.1107/S1600536814016547/lh5717sup1.cif

Structure factors: contains datablock(s) I. DOI: 10.1107/S1600536814016547/lh5717Isup2.hkl

Supporting information file. DOI: 10.1107/S1600536814016547/lh5717Isup3.cml

**Fig. 1.**
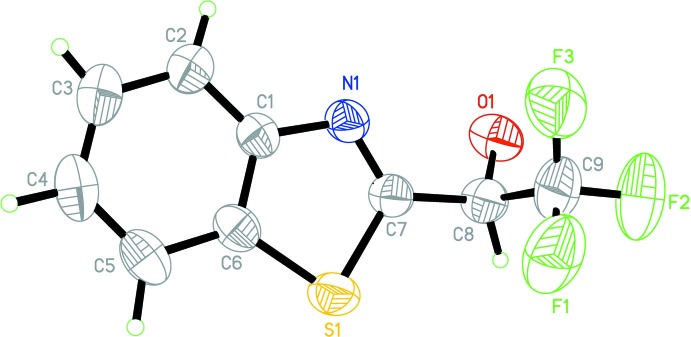
The molecular structure of the title compound with displacement ellipsoids drawn at the 50% probability level.

. DOI: 10.1107/S1600536814016547/lh5717fig1.tif
The mol­ecular structure of the title compound with displacement ellipsoids drawn at the 50% probability level.

**Fig. 2.**
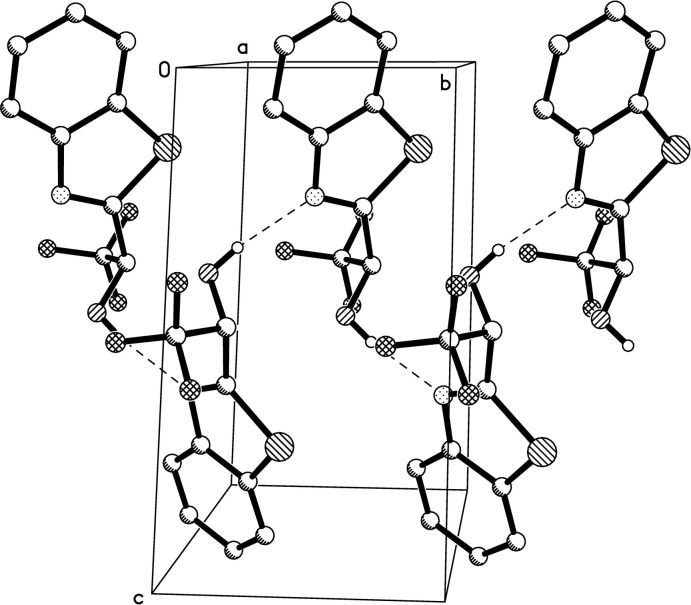
Part of the crystal structure with hydrogen bonds shown by dashed lines. Only H atoms involved in H-bonds are shown.

. DOI: 10.1107/S1600536814016547/lh5717fig2.tif
Part of the crystal structure with hydrogen bonds shown by dashed lines. Only H atoms involved in H-bonds are shown.

CCDC reference: 1014380

Additional supporting information:  crystallographic information; 3D view; checkCIF report

## Figures and Tables

**Table 1:** Hydrogen-bond geometry (Å, °)

*D*—H⋯*A*	*D*—H	H⋯*A*	*D*⋯*A*	*D*—H⋯*A*
O1—H1*O*⋯N1^i^	0.84 (4)	1.96 (4)	2.781 (2)	166 (4)

Symmetry code: (i) 
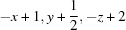
.

## supplementary crystallographic information

### S1. Comment

2,2,2-Trifluoro-1-substituted ethanols attract attention as building blocks for introducing a chiral CF_3_-containing motif into biologically active molecules and mimicking carboxylic groups. Among them, 2,2,2-trifluoro-1-heteroaryl ethanols, promising synthetic targets, have been poorly explored because of a lack of suitable procedures for obtaining the enantipure compounds from racemates. We have recently proposed a convenient procedure for enzyme-catalyzed kinetic resolution of racemic 2,2,2-trifluoro-1-heteroaryl ethanols on a series of 14 compounds. Herein, we report the crystal structure of (*S*)-1-(benzo[*d*]thiazol-2-yl)-2,2,2-trifluoroethanol (I) (Fig. 1). The non-centrosymmetric space group clearly confirms the presence of one enantiomer in the crystal. The absolute configuration of the chiral center at atom C8 (S-configuration) is determined using the value of the Flack parameter (-0.03 (9)). The substituent on the bicyclic fragment is oriented in such way that the hydroxyl group has a conformation intermediate between *sp-* and *-sc-* relative to the N1—C7 endocyclic bond (the N1—C7—C8—O1 torsion angle is -30.8 (3) °). The trifluoromethyl group is oriented in such way that the C9—F2 bond is *anti*-periplanar to the C7—C8 bond (the N1—C7—C8—C9 and C7—C8—C9—F2 torsion angles are 88.5 (3) ° and 177.3 (2) °, respectively). In the crystal, molecules are linked via O—H···N hydrogen bonds (Fig. 2) forming zigzag chains along [0 1 0].

### S2. Experimental

Synthesis of*rac*-1-(benzo[*d*]thiazol-2-yl)-2,2,2-trifluoroethanol: To a solution of 1-(benzo[*d*]thiazol-2-yl)-2,2,2-trifluoroethanone (115.5 g, 0.5 mol) in methanol (500 ml) sodium borohydride (18.9 g, 0.5 mol) was added in small portions, maintaining the temperature of the reaction mixture below 303K. The mixture was stirred at room temperature until completion of the reaction (monitored by TLC). The solvent was removed under reduced pressure; to the crude was added 200 ml of water and the aqueous solution was extracted with dichloromethane (3 × 150 ml). The organic phase was dried over Na_2_SO_4_ and evaporated yield the desired product. Yield: 114.2 g, 98%; white solid; m.p.: 377 K; ^1^H NMR (400 MHz, CDCl_3_): δ_H_ = 5.19 (qd, 1H, ^3^J_F,H_ = 7 Hz, ^3^J_H,H_ = 7 Hz, CH), 6.98–7.08 (m, 2H, PhH), 7.48 (d, 1H, ^3^J_H,H_ = 7 Hz, OH), 7.56 (d, 1H, ^3^J_H,H_ = 7.6 Hz, PhH), 7.66 (d, 1H, ^3^J_H,H_ = 8 Hz, PhH); ^13^C NMR (125 MHz, APT, CDCl_3_): δ_C_ = 69.4 (q, ^2^J_F,_*_C_* = 32 Hz, CH), 122.4 (PhH), 123.1 (PhH), 123.7 (q, ^1^J_F,_*_C_* = 282 Hz, CF_3_), 125.7 (PhH), 126.4 (PhH), 134.4 (C Ar), 152.7 (C Ar), 167.7 (C Ar); MS (APCI) m/*z* calculated for C_9_H_7_F_3_NOS 234.0 [*M*+H]^+^, found 234.0.

Kinetic resolution of*rac*-1-(benzo[*d*]thiazol-2-yl)-2,2,2-trifluoroethanol with vinyl acetate and*Burkholderia cepacia lipase*: The racemic alcohol (11.4 g, 0.05 mol) and vinyl acetate (14.3 ml, 0.15 mol) were dissolved in TBME (250 ml) following by addition of *Burkholderia cepacia* lipase (6 g). The obtained mixture was incubated at 323 K, the progress of the reaction was monitored by the cinchonidine method (Kolodyazhnyi *et al.*, 2006). Then, the enzyme was filtered off, washed with TBME and the combined TBME fractions were evaporated. The unacylated (*S*)-alcohol was separated from the (*R*)-ester by column chromatography (SiO_2_, eluent: AcOEt/hexanes gradually changed from 1:20 to 1:1 (*v*/*v*)). The white needle-like crystals of the (*S*)-alcohol were formed after 1 week upon crystallization from chloroform.

### S3. Refinement

The C—F bond lengths were constrained to 1.340 (1)Å. All hydrogen atoms were located in electron density difference maps and were refined with isotropic displacement parameters [C—H = 0.88 (4)–1.04 (3) Å and O—H = 0.84 (4)Å].

### Crystal data

**Table:**

C_9_H_6_F_3_NOS	*F*(000) = 236
*M**_r_* = 233.21	*D*_x_ = 1.565 Mg m^−^^3^
Monoclinic, *P*2_1_	Mo *K*α radiation, λ = 0.71073 Å
Hall symbol: P 2yb	Cell parameters from 1591 reflections
*a* = 9.2116 (9) Å	θ = 3.5–31.8°
*b* = 5.5052 (4) Å	µ = 0.34 mm^−^^1^
*c* = 10.2279 (8) Å	*T* = 293 K
β = 107.411 (9)°	Needle, colourless
*V* = 494.91 (7) Å^3^	0.20 × 0.05 × 0.05 mm
*Z* = 2	

### Data collection

**Table:**

Agilent Xcalibur3 diffractometer	2768 independent reflections
Radiation source: Enhance (Mo) X-ray Source	2293 reflections with *I* > 2σ(*I*)
Graphite monochromator	*R*_int_ = 0.027
Detector resolution: 16.1827 pixels mm^-1^	θ_max_ = 30.0°, θ_min_ = 3.6°
ω scans	*h* = −10→12
Absorption correction: multi-scan (*CrysAlis RED*; Agilent, 2012)	*k* = −7→7
*T*_min_ = 0.935, *T*_max_ = 0.983	*l* = −13→14
4650 measured reflections	

### Refinement

**Table:**

Refinement on *F*^2^	Secondary atom site location: difference Fourier map
Least-squares matrix: full	Hydrogen site location: difference Fourier map
*R*[*F*^2^ > 2σ(*F*^2^)] = 0.042	All H-atom parameters refined
*wR*(*F*^2^) = 0.108	*w* = 1/[σ^2^(*F*_o_^2^) + (0.0494*P*)^2^] where *P* = (*F*_o_^2^ + 2*F*_c_^2^)/3
*S* = 1.12	(Δ/σ)_max_ < 0.001
2768 reflections	Δρ_max_ = 0.21 e Å^−^^3^
160 parameters	Δρ_min_ = −0.20 e Å^−^^3^
4 restraints	Absolute structure: Flack (1983), 1199 Friedel pairs
Primary atom site location: structure-invariant direct methods	Absolute structure parameter: −0.03 (9)

### Special details

**Table:**

Geometry. All e.s.d.'s (except the e.s.d. in the dihedral angle between two l.s. planes) are estimated using the full covariance matrix. The cell e.s.d.'s are taken into account individually in the estimation of e.s.d.'s in distances, angles and torsion angles; correlations between e.s.d.'s in cell parameters are only used when they are defined by crystal symmetry. An approximate (isotropic) treatment of cell e.s.d.'s is used for estimating e.s.d.'s involving l.s. planes.
Refinement. Refinement of *F*^2^ against ALL reflections. The weighted *R*-factor *wR* and goodness of fit *S* are based on *F*^2^, conventional *R*-factors *R* are based on *F*, with *F* set to zero for negative *F*^2^. The threshold expression of *F*^2^ > σ(*F*^2^) is used only for calculating *R*-factors(gt) *etc*. and is not relevant to the choice of reflections for refinement. *R*-factors based on *F*^2^ are statistically about twice as large as those based on *F*, and *R*- factors based on ALL data will be even larger.

### Fractional atomic coordinates and isotropic or equivalent isotropic displacement parameters (Å^2^)

**Table:**

	*x*	*y*	*z*	*U*_iso_*/*U*_eq_	
F1	0.84725 (19)	0.5557 (5)	0.85264 (17)	0.0991 (7)	
F2	0.89881 (19)	0.5084 (6)	1.06943 (16)	0.1122 (8)	
F3	0.7640 (2)	0.2434 (2)	0.9323 (2)	0.0913 (6)	
S1	0.51635 (7)	0.81353 (17)	0.68465 (5)	0.05600 (17)	
N1	0.4284 (2)	0.4285 (3)	0.78357 (16)	0.0454 (4)	
O1	0.5967 (2)	0.5338 (3)	1.05476 (15)	0.0604 (4)	
H1O	0.582 (4)	0.637 (7)	1.110 (3)	0.088 (11)*	
C1	0.3304 (2)	0.4556 (4)	0.65070 (19)	0.0434 (4)	
C2	0.2101 (3)	0.3050 (6)	0.5878 (2)	0.0579 (5)	
H2	0.189 (3)	0.176 (5)	0.643 (2)	0.046 (6)*	
C3	0.1234 (3)	0.3593 (6)	0.4559 (3)	0.0651 (7)	
H3	0.050 (4)	0.253 (7)	0.424 (3)	0.089 (11)*	
C4	0.1525 (3)	0.5594 (6)	0.3878 (2)	0.0634 (7)	
H4	0.093 (3)	0.582 (6)	0.293 (3)	0.067 (8)*	
C5	0.2713 (3)	0.7138 (5)	0.4479 (2)	0.0569 (6)	
H5	0.299 (3)	0.864 (7)	0.408 (3)	0.072 (9)*	
C6	0.3606 (3)	0.6586 (4)	0.5814 (2)	0.0463 (5)	
C7	0.5280 (3)	0.6011 (4)	0.81243 (18)	0.0440 (4)	
C8	0.6483 (3)	0.6243 (5)	0.9503 (2)	0.0511 (5)	
H8	0.689 (2)	0.799 (5)	0.976 (2)	0.044 (5)*	
C9	0.7885 (2)	0.4822 (3)	0.95081 (14)	0.0689 (8)	

### Atomic displacement parameters (Å^2^)

**Table:**

	*U*^11^	*U*^22^	*U*^33^	*U*^12^	*U*^13^	*U*^23^
F1	0.0638 (10)	0.153 (2)	0.0853 (11)	−0.0105 (12)	0.0298 (9)	−0.0049 (12)
F2	0.0743 (11)	0.165 (2)	0.0710 (10)	0.0026 (14)	−0.0187 (9)	−0.0216 (13)
F3	0.0854 (12)	0.0762 (13)	0.1038 (13)	0.0216 (10)	0.0154 (10)	−0.0137 (9)
S1	0.0703 (3)	0.0513 (3)	0.0463 (3)	−0.0111 (3)	0.0173 (2)	0.0057 (2)
N1	0.0535 (10)	0.0439 (9)	0.0372 (8)	−0.0022 (8)	0.0111 (7)	0.0016 (6)
O1	0.0912 (13)	0.0530 (10)	0.0380 (7)	−0.0013 (9)	0.0208 (7)	−0.0040 (7)
C1	0.0462 (10)	0.0442 (10)	0.0392 (9)	0.0031 (8)	0.0120 (7)	0.0018 (8)
C2	0.0510 (11)	0.0607 (13)	0.0571 (12)	−0.0075 (13)	0.0088 (9)	0.0033 (13)
C3	0.0495 (13)	0.078 (2)	0.0591 (13)	0.0007 (13)	0.0028 (10)	−0.0055 (13)
C4	0.0573 (13)	0.0851 (19)	0.0413 (11)	0.0148 (13)	0.0047 (9)	−0.0017 (11)
C5	0.0678 (15)	0.0633 (14)	0.0416 (11)	0.0140 (12)	0.0195 (10)	0.0101 (10)
C6	0.0545 (12)	0.0476 (11)	0.0392 (9)	0.0058 (9)	0.0177 (8)	0.0028 (8)
C7	0.0532 (11)	0.0427 (10)	0.0363 (9)	−0.0023 (9)	0.0137 (8)	−0.0020 (7)
C8	0.0655 (14)	0.0476 (12)	0.0369 (10)	−0.0071 (10)	0.0102 (9)	−0.0073 (8)
C9	0.0584 (15)	0.089 (2)	0.0496 (13)	−0.0059 (15)	0.0011 (10)	−0.0111 (13)

### Geometric parameters (Å, º)

**Table:**

F1—C9	1.3382 (10)	C2—C3	1.379 (3)
F2—C9	1.3372 (10)	C2—H2	0.96 (3)
F3—C9	1.3376 (10)	C3—C4	1.372 (4)
S1—C6	1.731 (2)	C3—H3	0.88 (4)
S1—C7	1.733 (2)	C4—C5	1.377 (4)
N1—C7	1.292 (3)	C4—H4	0.96 (3)
N1—C1	1.396 (2)	C5—C6	1.400 (3)
O1—C8	1.385 (3)	C5—H5	0.99 (4)
O1—H1O	0.84 (4)	C7—C8	1.516 (3)
C1—C2	1.379 (3)	C8—C9	1.509 (3)
C1—C6	1.395 (3)	C8—H8	1.04 (3)
			
C6—S1—C7	88.89 (10)	C1—C6—C5	121.4 (2)
C7—N1—C1	110.59 (17)	C1—C6—S1	109.85 (15)
C8—O1—H1O	116 (3)	C5—C6—S1	128.7 (2)
C2—C1—C6	119.94 (19)	N1—C7—C8	123.09 (18)
C2—C1—N1	125.8 (2)	N1—C7—S1	116.39 (14)
C6—C1—N1	114.27 (18)	C8—C7—S1	120.51 (17)
C1—C2—C3	118.3 (3)	O1—C8—C9	107.55 (18)
C1—C2—H2	116.4 (13)	O1—C8—C7	111.31 (19)
C3—C2—H2	125.0 (14)	C9—C8—C7	110.22 (16)
C4—C3—C2	121.8 (3)	O1—C8—H8	108.9 (11)
C4—C3—H3	126 (2)	C9—C8—H8	103.3 (13)
C2—C3—H3	112 (2)	C7—C8—H8	115.0 (12)
C3—C4—C5	121.3 (2)	F2—C9—F3	106.5 (2)
C3—C4—H4	118.2 (19)	F2—C9—F1	106.24 (18)
C5—C4—H4	120.3 (18)	F3—C9—F1	106.3 (2)
C4—C5—C6	117.2 (2)	F2—C9—C8	111.38 (19)
C4—C5—H5	127.0 (15)	F3—C9—C8	113.65 (17)
C6—C5—H5	115.7 (16)	F1—C9—C8	112.25 (18)
			
C7—N1—C1—C2	−179.2 (2)	C1—N1—C7—C8	178.7 (2)
C7—N1—C1—C6	−0.5 (3)	C1—N1—C7—S1	−0.2 (2)
C6—C1—C2—C3	0.8 (4)	C6—S1—C7—N1	0.62 (18)
N1—C1—C2—C3	179.4 (2)	C6—S1—C7—C8	−178.34 (19)
C1—C2—C3—C4	−1.1 (4)	N1—C7—C8—O1	−30.8 (3)
C2—C3—C4—C5	0.9 (4)	S1—C7—C8—O1	148.13 (17)
C3—C4—C5—C6	−0.3 (4)	N1—C7—C8—C9	88.5 (3)
C2—C1—C6—C5	−0.2 (3)	S1—C7—C8—C9	−92.6 (2)
N1—C1—C6—C5	−179.1 (2)	O1—C8—C9—F2	−61.2 (2)
C2—C1—C6—S1	179.74 (19)	C7—C8—C9—F2	177.3 (2)
N1—C1—C6—S1	0.9 (2)	O1—C8—C9—F3	59.1 (2)
C4—C5—C6—C1	0.0 (4)	C7—C8—C9—F3	−62.4 (2)
C4—C5—C6—S1	−180.0 (2)	O1—C8—C9—F1	179.82 (18)
C7—S1—C6—C1	−0.83 (16)	C7—C8—C9—F1	58.3 (2)
C7—S1—C6—C5	179.1 (2)		

### Hydrogen-bond geometry (Å, º)

**Table:**

*D*—H···*A*	*D*—H	H···*A*	*D*···*A*	*D*—H···*A*
O1—H1*O*···N1^i^	0.84 (4)	1.96 (4)	2.781 (2)	166 (4)

Symmetry code: (i) −*x*+1, *y*+1/2, −*z*+2.
