# Supplementary figures and images for: Crystal structure of (S)-1-(1,3-benzo­thia­zol-2-yl)-2,2,2-tri­fluoro­ethanol
Source: Acta Crystallogr Sect E Struct Rep Online. 2014 Aug 1;70(Pt 9):o946. doi: 10.1107/S1600536814016547 (PMC4186080; doi:10.1107/S1600536814016547)

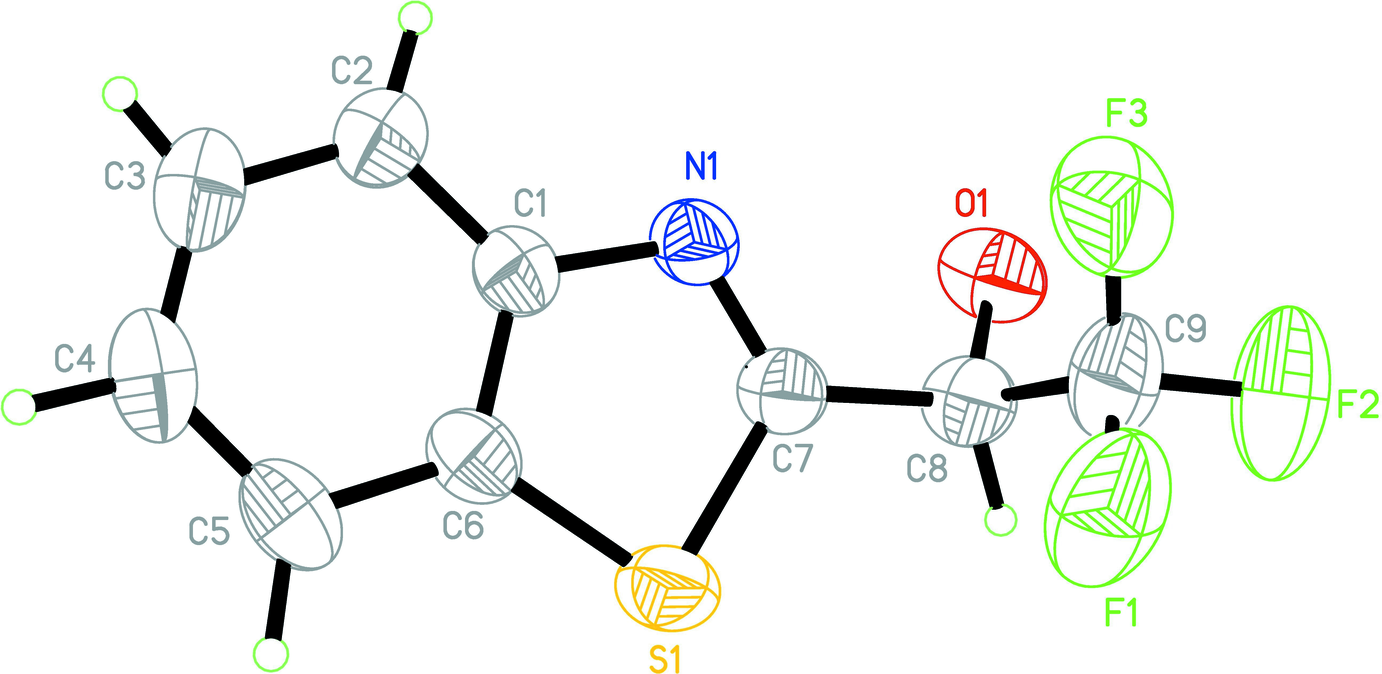

Supplement: Supplementary file 4 [file e-70-0o946-fig1.tif]

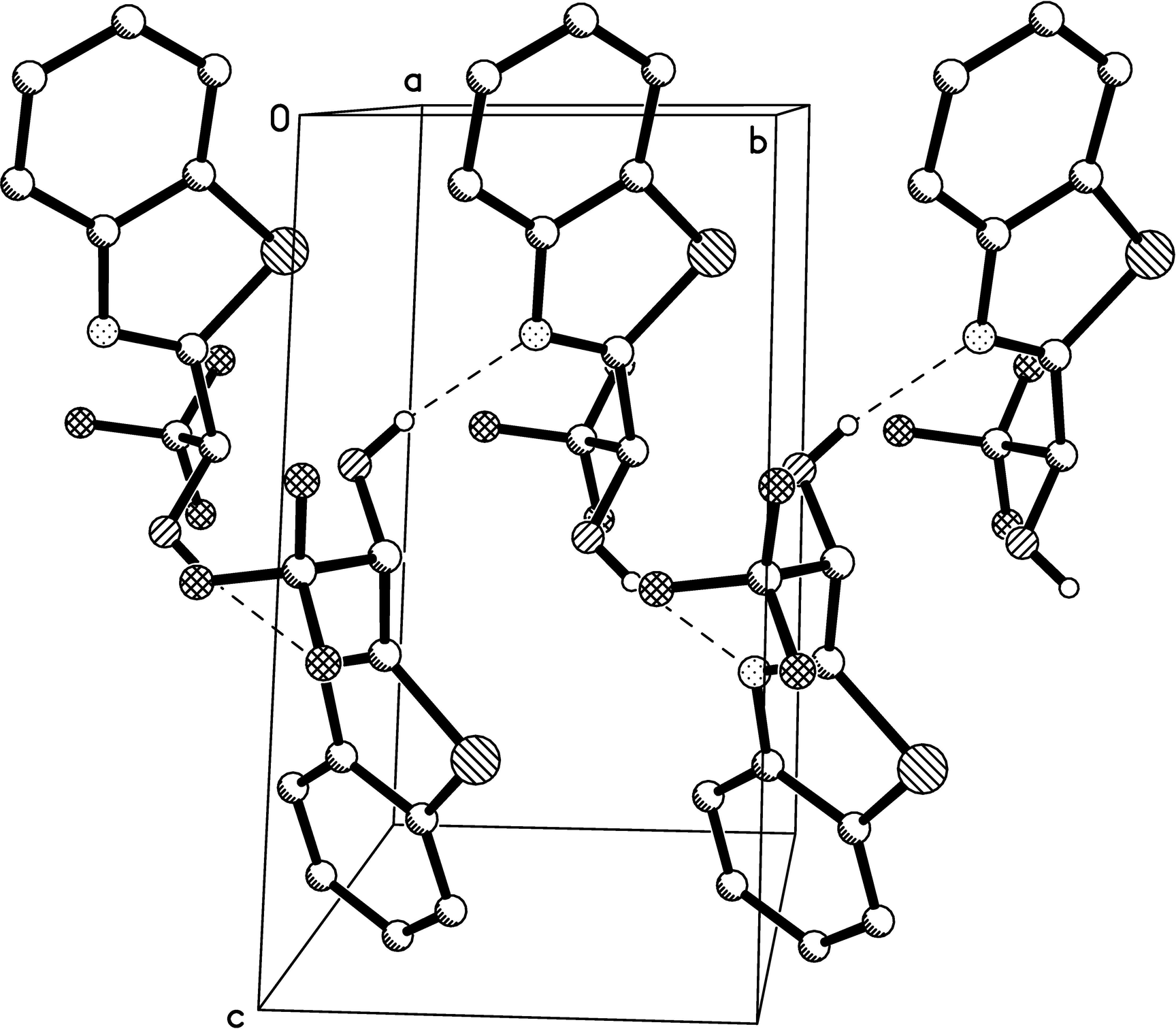

Supplement: Supplementary file 5 [file e-70-0o946-fig2.tif]
